# Effective visualization of biomedical data using *plot-misc*

**DOI:** 10.1093/bioadv/vbag184

**Published:** 2026-06-27

**Authors:** Amand Floriaan Schmidt, Nikita Hukerikar, Chris Finan, Marion van Vugt

**Affiliations:** Institute of Cardiovascular Science, Faculty of Population Health, University College London, London WC1E 6HX, United Kingdom; University College London, British Heart Foundation Center of Excellence, London WC1E 6HX, United Kingdom; Department of Cardiology, Amsterdam Cardiovascular Sciences, Amsterdam University Medical Centres, University of Amsterdam, Amsterdam UMC, Amsterdam Zuidoost 1100 DD, The Netherlands; Institute of Cardiovascular Science, Faculty of Population Health, University College London, London WC1E 6HX, United Kingdom; University College London, British Heart Foundation Center of Excellence, London WC1E 6HX, United Kingdom; Institute of Cardiovascular Science, Faculty of Population Health, University College London, London WC1E 6HX, United Kingdom; University College London, British Heart Foundation Center of Excellence, London WC1E 6HX, United Kingdom; Institute of Cardiovascular Science, Faculty of Population Health, University College London, London WC1E 6HX, United Kingdom; University College London, British Heart Foundation Center of Excellence, London WC1E 6HX, United Kingdom; Department of Cardiology, Amsterdam Cardiovascular Sciences, Amsterdam University Medical Centres, University of Amsterdam, Amsterdam UMC, Amsterdam Zuidoost 1100 DD, The Netherlands

## Abstract

**Motivation:**

Computational visualizations are prevalent in all disciplines of biomedical science, from wet-lab research, through clinical science to population health and epidemiology. While *matplotlib* and *seaborn* are established Python tools for generating illustrations, both omit visualization archetypes commonly used in biomedical research. Producing such visualization using *matplotlib’s* low-level interface may result in verbose, brittle code that demands considerable programming experience, and limits reuse between projects and users.

**Results:**

*plot-misc* is a Python package that is designed specifically for publication quality biomedical research visualization. It combines fine-grained control with archetype-based plotting, prioritizing customizable figure generation over integrated statistical routines. Available archetypes include forest plots, survival plots, volcano plots, heatmaps, and incidence matrices. Thanks to its *matplotlib*-first design, most Python users will be able to readily integrate *plot-misc* into existing routines. Online tutorials are available to onboard new users and provide robust code examples. *Plot-misc* provides a unified and flexible framework for creating high-quality, publication-ready illustrations that caters to the diverse visualization needs of modern biomedical researchers.

**Availability and implementation:**

*plot-misc* is available on Conda, PyPi, as well as through GitLab: https://schmidtaf.gitlab.io/plot-misc.

## 1 Introduction

Biomedical research spans a wide range of domains, including wet-lab, genetics and other types of omics, as well as clinical and population-based health research. As these domains have grown in scale and complexity, so too have the tools required to effectively visualize outputs in presentations and publications. Solutions for generating publication-quality illustrations must provide simple and intuitive application programming interfaces (API), while remaining sufficiently customizable to cater to the diverse needs of biomedical researchers with varying levels of computational expertise.

Python is one of the leading programming languages used in biomedical research, where plotting libraries such as *matplotlib* (Hunter 2007) and *seaborn* (Waskom 2021) are established tools for creating (static) illustrations. *matplotlib* is a versatile low-level package providing users with fine-grained control over individual figure elements. *seaborn*, which is built on top of *matplotlib*, provides a higher level of abstraction, and introduces plotting archetypes based on their function (e.g. distributional, categorical, or relational). Although *matplotlib’*s low-level API allows a user to create a wide range of static illustrations, the lack of higher-level plotting archetypes may lead to verbose and brittle code, which often needs to be expanded further to create publication-ready illustrations. In contrast, the plotting archetypes offered by *seaborn* overlook common plotting types used in biomedical research (e.g. volcano plots or forest plots). Furthermore, by intertwining plotting with statistical analysis, *seaborn* introduces analytical constraints which may limit customization.

We have developed a Python-based package, *plot-misc*, which specifically focuses on visualization types used commonly in biomedical research, such as forest plots, volcano plots, survival plots, and machine learning illustrations, which are currently poorly supported by existing Python libraries; please refer to Table 1 for an overview of plot archetypes. *plot-misc* marries the flexibility of matplotlib with the plotting archetype framework popularized by seaborn, while deliberately prioritizing visualization over integrating statistical functionalities. As every callable returns matplotlib Figure and Axes objects, the package is fully interoperable with matplotlib. By abstracting away much of the complexity of native matplotlib artist-focused plotting, the user can instead focus on the customizations necessary to create publication ready illustration. In doing so, *plot-misc* provides a consistent framework that reduces code complexity and improves reproducibility and reusability.

**Table 1:** A TLDR overview of the main plotting types in *plot-misc*.^a^

Archetype	Main function/class	Example illustration
Bar chart	# simple bar chart bar() # grouped bar chart group_bar() # stacked bar chart stack_bar() # sub/total bar chart subtotal_bar()	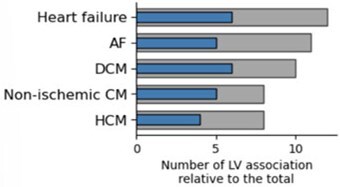
Bubble chart	draw_incidencematrix( ..,grid_position=“outline”)	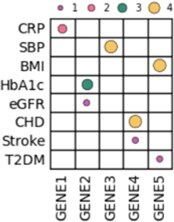
Forest plot	ForestPlot.plot() # optional side table plot_table()	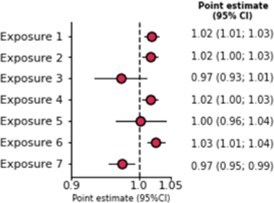
Annotated heatmap	# simple heatmap heatmap() # optional annotation annotate_heatmap() # optional preprocessing utils.calc_matrices()	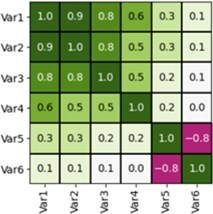
Incidence matrix	draw_incidencematrix( ..,grid_position=“ centre”)	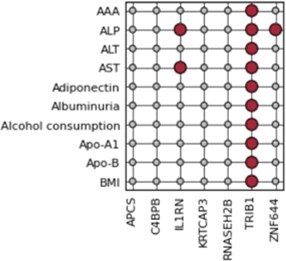
Machine learning plots	# calibration plot Calibration() # lollipop plot lollipop() # calculate net benefit DecisionCurve.calc_net_benefit() # decision curve plot DecisionCurve.plot()	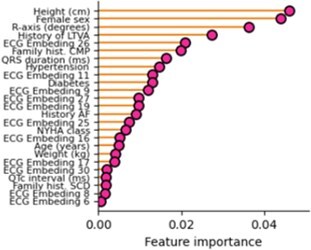
Annotated pie chart	piechart()	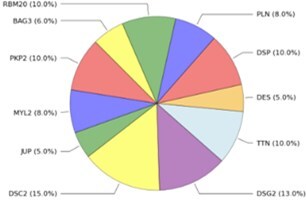
Survival plot	# survival plot plot_step_wise() # optional bottom table plot_table() # optional preprocessing extract_follow_up()	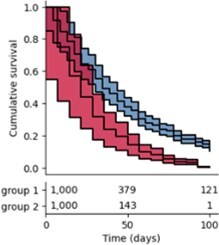
Tree/compatibility plot	EmpiricalSupport.plot_tree()	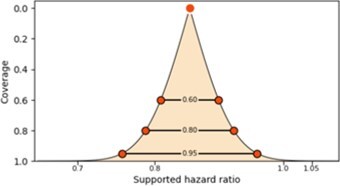
Volcano plot	plot_volcano()	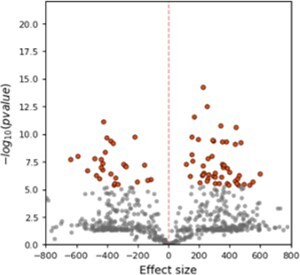

a Please refer to the online documentation for worked out examples as well as the full API documentation: https://schmidtaf.gitlab.io/plot-misc/.

## 2 Results

### 2.1 Supported plotting types and design philosophy

The package is developed with a focus on generating high quality illustrations. As such, it is expected that input data have been curated and appropriately formatted in advance. This ensures a transparent relationship between input data and visualizations, while supporting user control over the analytical input. For example, rather than integrating dedicated statistical routines for confidence interval estimation (e.g. based on a Wald approximation, or using the test inversion principle), user derived estimates can be directly supplied. In cases where plotting is tightly coupled with analytical steps, such as plot types that canonically include line smoothing, the library supports user customizability. This is achieved through function parameters which accept (user defined) callable objects (i.e. a code object that can be invoked or executed) to control data transformation, and by exposing internally generated data for reuse or inspection.

**Figure 1:**
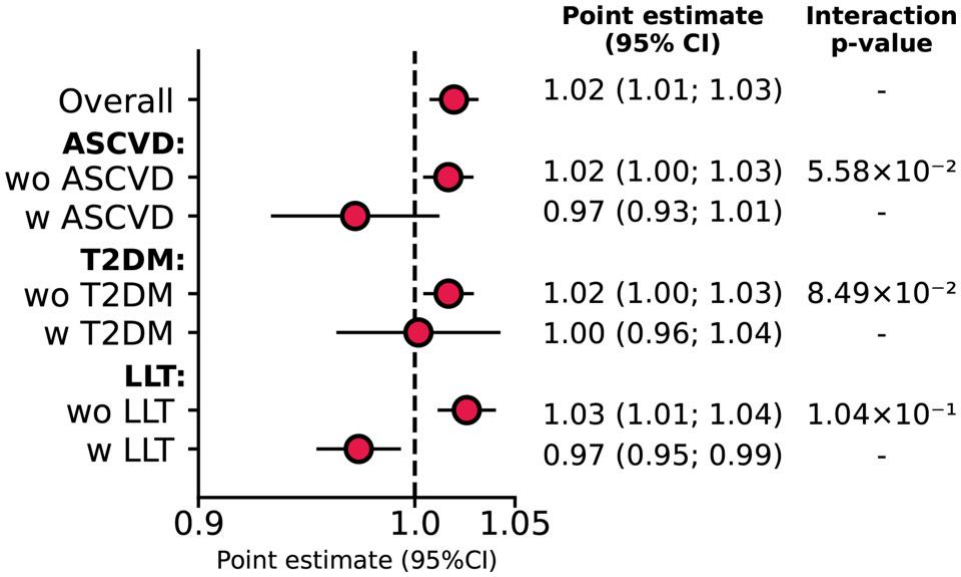
Forest plot with side table created using *plot-misc*. Point estimates are presented as dots with lines used to indicate 95% confidence intervals, with the vertical line representing the absence of an association. The overall estimate was stratified by the baseline variables: atherosclerotic cardiovascular disease (ASCVD), type 2 diabetes (T2DM), and lipid lowering treatment (LLT).

The *matplotlib*-first principle of *plot-misc* is illustrated when considering forest plots and survival plots, which typically combine two distinct display types: graphical elements (lines/point) and formatted text. In *plot-misc* this is addressed by providing separate functionality for each display type, designed to render on distinct, adjacent Axes objects; Fig. 1, Code example 1. This separation avoids the artefacts and layout issues that can arise when heterogeneous display elements are rendered on a shared axis.

**Code example 1**. Using *plot-misc* to generate a forest plot.

import matplotlib.pyplot as plt

import plot_misc.forest as forest

from matplotlib.gridspec import GridSpec

# Create two axes

CMTOINCH = 1/2.54

fig = plt.figure(figsize=

  (6*CMTOINCH, 5*CMTOINCH))

gs = GridSpec(nrows = 1, ncols = 2,

  width_ratios=[6, 4],

  figure=fig)

ax_main = fig.add_subplot(gs[(0,0)])

ax_table = fig.add_subplot(gs[(0,1)])

# Create a forest plot

# NOTE: V, P, L, U are columns in table

table = forest.set_y_coordinates(table,

  group='V')

trees = forest. ForestPlot(

  data=table, x_col='P', lb_col='L',

  ub_col='U',g_col='V', ax=ax_main,

)

trees.plot(

  c_col='col’, s_size_col = 40,

  ci_lwd = 0.8, ci_colour='black’,

  kwargs_plot_ci_dict={'zorder’:1},

)

# Create side table

# NOTE: S is a column in table

# containing formatted strings

x_table.set_ylim(ax_main.get_ylim())

_ = forest.plot_table(

  table,

  annoteheader=\

     Point estimate\n(95% CI)',

  string_col=S,size_text = 7,

  size_header = 7, negative_padding = 1,

  ax=ax_table,

)

# Next use standard matplotlib to customize the illustration.

**Figure 2:**
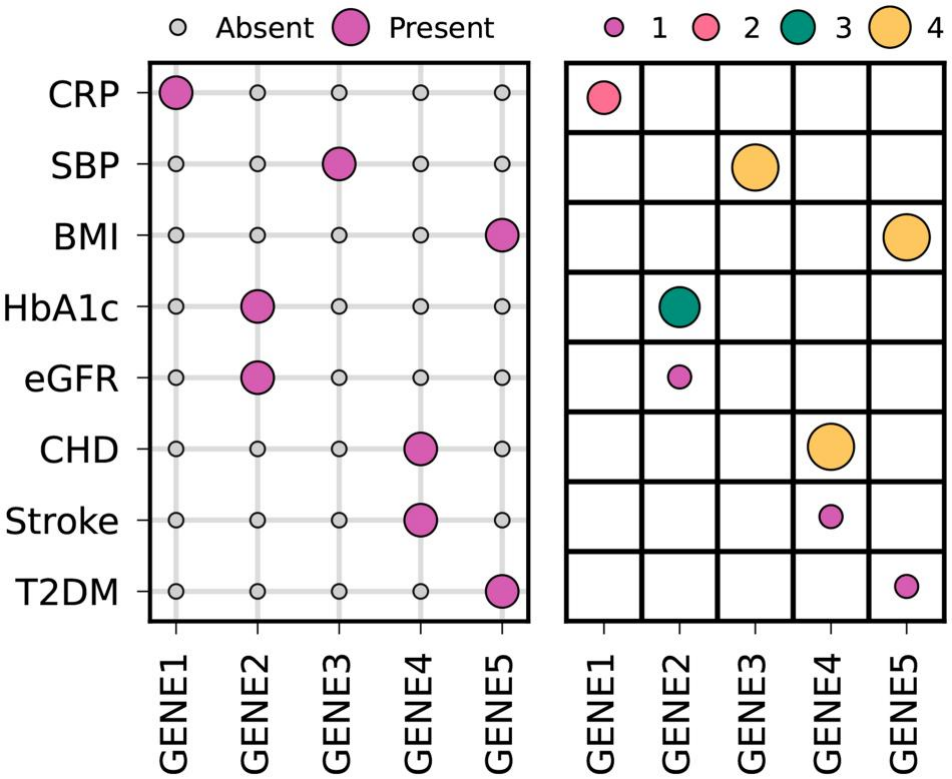
Incidence matrix and bubble chart created using *plot-misc*. The incidence matrix depicts pairwise relationships between the *y*-axis traits and any variant mapped to genes listed on the *x*-axis. The left-hand side bubble chart depicts the same data but now accounting for the number of variants (represented as size and color) associated with the listed trait.

Where practical, *plot-misc* combines plotting variants into a single parameterized function. For example, bar charts and lollipop plots can be orientated both horizontally and vertically without the need to use a distinct function. As the package relies on the user to supply pre-calculated input data, the functionality can remain type-agnostic, and count and proportion data can be rendered using the same underlying callable (contrary to e.g. *seaborn*). Similarly, the incidence matrix function can be used to illustrate incidence matrices (i.e. representing one-or-more pairwise connections) as well as used to create bubble charts (i.e. a dot based categorical heatmap); Fig. 2 and Table 1

### 2.2 Comparison against alternative packages

Compared to existing Python forest plot implementations such as *forestplot* (Shen 2024), which offers complex publication ready illustrations, our implementation emphasizes customizability. *plot-misc* provides users with the tools to create similar outputs without enforcing any specific style or format, and while maintaining a clear separation between text and graphical elements; see Code example 1. The survival plot functionality provided by *plot-misc* is similar to that offered by the *lifelines* (Davidson-Pilon 2019) package, with the important difference that, rather than working from an instance of a callable, *plot-misc* operates directly on tabular data. This design choice ensures that analysis and plotting are strictly separated, supporting scaled analysis where direct visual fine tuning is unwarranted (or when running remote analyses). Compared to the *dcurves* package (Porwal and Singh 2025), which provides a Python port of the *dcurves* R package, *plot-misc* offers similar illustrations with increased customization and a more direct interface with matplotlib. The Supplementary Code, available as supplementary data at *Bioinformatics Advances* online, further illustrates the reduction in code complexity by comparing a pure *matplotlib* implementation of Fig. 2 with the equivalent *plot-misc* version.

### 2.3 Implementation and distribution

*plot-misc* is implemented with a complete set of unit tests and (where relevant) end-to-end tests, as well as GitLab-based continuous integration and deployment. The package is available through Conda as well as PyPi. Sphinx is used to provide detailed and automatically updated documentation, including quick start plotting recipes using Jupyter notebooks; Table 1.

## 3 Discussion

Biomedical research is conducted in a vast number of settings, where the resulting data often requires bespoke visualizations. Together with the increasing integration of computational workflows into day-to-day research practice, there is a clear need for a Python package providing plotting archetypes relevant for biomedical research, reducing the need for custom code generation, decreasing complexity, and improving reproducibility.

By focusing on common biomedical visualization archetypes (Table 1), *plot-misc* abstracts recurring visual structures into reusable components. While archetypical implementation provides meaningful reductions in code complexity, generating publication-ready illustrations will always require further (user-specific) customization. To address this, *plot-misc* is fully interoperable with *matplotlib*, which adopts an *artist-first* approach, allowing appropriate control over individual plot elements such as lines, markers, and shapes.

Rather than developing a unique API, which may be time-consuming to learn, the *matplotlib*-first implementation of *plot-misc* allows researchers already familiar with its API to integrate the package into existing codebases without learning a new approach. To reduce implementation time further, the package ships with extensive documentation. The documentation is also valuable as context for coding support from large language models, where well-structured, tested archetypes reduce the prompting, iterations, and tokens needed to produce reliable biomedical illustrations.

In conclusion, *plot-misc* provides a flexible visualization framework for biomedical research. By combining a uniform interface with full customizability and a clear separation between analysis and visualization, it supports publication-quality figure generation across diverse computational workflows encountered in biomedical settings.

## Supplementary Material

vbag184_Supplementary_Data

## Data Availability

*plot-misc* is available under a GPL-3.0-or-later license, please see https://gitlab.com/SchmidtAF/plot-misc for the publicly available GitLab repository which can also be used to raise issues. Future releases will be additionally published on conda (https://anaconda.org/channels/afschmidt/packages/plot-misc/) and PyPi (https://pypi.org/project/plot-misc/).
