# Supplementary material for: Effective visualization of biomedical data using plot-misc
Source: Bioinform Adv. 2026 Jun 27;6(1):vbag184. doi: 10.1093/bioadv/vbag184 (PMC13412160; doi:10.1093/bioadv/vbag184)
Supplement: vbag184_Supplementary_Data [file vbag184_supplementary_data.docx]

**Supplementary code to reproduce manuscript Figure 2 using matplotlib only, compared to using plot-misc.**

Data generation pre-amble.

import numpy as np

import pandas as pd

# ---------------------------------------------------------------------------

# example data

table = pd.DataFrame(

np.zeros((8, 5), dtype=int),

columns=['GENE1', 'GENE2', 'GENE3', 'GENE4', 'GENE5'],

index=['CRP', 'SBP', 'BMI', 'HbA1c', 'eGFR', 'CHD',

'Stroke', 'T2DM'],

)

table.loc['SBP', 'GENE3'] = 4

table.loc['CHD', 'GENE4'] = 4

table.loc['HbA1c', 'GENE2'] = 3

table.loc['BMI', 'GENE5'] = 4

table.loc['CRP', 'GENE1'] = 2

table.loc['eGFR', 'GENE2'] = 1

table.loc['Stroke', 'GENE4'] = 1

table.loc['T2DM', 'GENE5'] = 1

Matplotlib only implementation (70 function calls, excluding example data generation)

import numpy as np

import pandas as pd

import matplotlib.pyplot as plt

from matplotlib.lines import Line2D

from matplotlib.patches import Patch

from matplotlib.gridspec import GridSpec

# ---------------------------------------------------------------------------

# Threshold rules.

BREAK_LOW = -np.inf

colour_rule_left = [('#C4C4C4', 0.0), ('#d65db1', np.inf)]

size_rule_left = [(8.0, 0.0), (40.0, np.inf)]

alpha_rule_left = [(0.9, 0.0), (1.0, np.inf)]

colour_rule_right = [

('#AAAAAA', 0.9),

('#d65db1', 1.9),

('#ff6f91', 2.9),

('#008f7a', 3.9),

('#ffc75f', 4.9),

]

size_rule_right = [

(0.0, 0.9),

(20.0, 1.9),

(40.0, 2.9),

(60.0, 3.9),

(80.0, 4.9),

]

alpha_rule_right = [

(1.0, 0.9),

(1.0, 1.9),

(1.0, 2.9),

(1.0, 3.9),

(1.0, 4.9),

]

data_left = (table > 0).astype(int).iloc[::-1].T

data_right = table.iloc[::-1].T

M, N = data_left.shape

vals_left = data_left.to_numpy()

vals_right = data_right.to_numpy()

# ---------------------------------------------------------------------------

# Map left-panel values to colours, sizes, and alphas.

mapped_colours_left = np.full_like(vals_left, np.nan, dtype=object)

cut_low = BREAK_LOW

for colour, cut_high in sorted(colour_rule_left, key=lambda rule: rule[1]):

selected = (vals_left > cut_low) & (vals_left <= cut_high)

mapped_colours_left[selected] = colour

cut_low = cut_high

mapped_sizes_left = np.full_like(vals_left, np.nan, dtype=float)

for size, cut_high in sorted(size_rule_left, key=lambda rule: rule[1]):

selected = (vals_left > cut_low) & (vals_left <= cut_high)

mapped_sizes_left[selected] = size

cut_low = cut_high

mapped_alphas_left = np.full_like(vals_left, np.nan, dtype=float)

for alpha, cut_high in sorted(alpha_rule_left, key=lambda rule: rule[1]):

selected = (vals_left > cut_low) & (vals_left <= cut_high)

mapped_alphas_left[selected] = alpha

cut_low = cut_high

# ---------------------------------------------------------------------------

# Map right-panel values to colours, sizes, and alphas using the same logic.

mapped_colours_right = np.full_like(vals_right, np.nan, dtype=object)

for colour, cut_high in sorted(colour_rule_right, key=lambda rule: rule[1]):

selected = (vals_right > cut_low) & (vals_right <= cut_high)

mapped_colours_right[selected] = colour

cut_low = cut_high

mapped_sizes_right = np.full_like(vals_right, np.nan, dtype=float)

for size, cut_high in sorted(size_rule_right, key=lambda rule: rule[1]):

selected = (vals_right > cut_low) & (vals_right <= cut_high)

mapped_sizes_right[selected] = size

cut_low = cut_high

mapped_alphas_right = np.full_like(vals_right, np.nan, dtype=float)

for alpha, cut_high in sorted(alpha_rule_right, key=lambda rule: rule[1]):

selected = (vals_right > cut_low) & (vals_right <= cut_high)

mapped_alphas_right[selected] = alpha

cut_low = cut_high

# ---------------------------------------------------------------------------

# Build the per-cell plotting coordinates.

x_coords = np.arange(M)

y_coords = np.arange(N)

x, y = np.meshgrid(x_coords, y_coords)

xv = x.T.ravel()

yv = y.T.ravel()

colour_flat_left = mapped_colours_left.ravel()

size_flat_left = mapped_sizes_left.ravel()

alpha_flat_left = mapped_alphas_left.ravel()

colour_flat_right = mapped_colours_right.ravel()

size_flat_right = mapped_sizes_right.ravel()

alpha_flat_right = mapped_alphas_right.ravel()

# ---------------------------------------------------------------------------

# Figure

CMTOINCH = 1 / 2.54

fig = plt.figure(figsize=(7 * CMTOINCH, 6 * CMTOINCH))

gs = GridSpec(

nrows=2, ncols=2, width_ratios=[1, 1], height_ratios=[1, 10],

wspace=0.1, figure=fig,

)

ax_l = fig.add_subplot(gs[1, 0])

ax_r = fig.add_subplot(gs[1, 1])

leg_ax = fig.add_subplot(gs[0, :])

leg_ax.axis('off')

# ---------------------------------------------------------------------------

# Left panel

for colour in np.unique(colour_flat_left):

mask = colour_flat_left == colour

if not np.any(mask):

continue

ax_l.scatter(

xv[mask], yv[mask],

s=size_flat_left[mask],

facecolor=colour,

edgecolor='black',

linewidths=0.4,

alpha=alpha_flat_left[mask],

zorder=3,

)

for x_line in np.arange(M):

ax_l.axvline(x=x_line, c='lightgrey', linestyle='-', linewidth=1.0,

zorder=1)

for y_line in np.arange(N):

ax_l.axhline(y=y_line, c='lightgrey', linestyle='-', linewidth=1.0,

zorder=1)

ax_l.set_xticks(np.arange(M))

ax_l.set_xticklabels(data_left.index, rotation=90)

ax_l.set_yticks(np.arange(N))

ax_l.set_yticklabels(data_left.columns)

ax_l.tick_params(axis='x', labelsize=7, length=2.5)

ax_l.tick_params(axis='y', labelsize=7, length=2.5)

ax_l.margins(x=0.08, y=0.06)

# ---------------------------------------------------------------------------

# Right panel

for colour in np.unique(colour_flat_right):

mask = colour_flat_right == colour

if not np.any(mask):

continue

ax_r.scatter(

xv[mask], yv[mask],

s=size_flat_right[mask],

facecolor=colour,

edgecolor='black',

linewidths=0.4,

alpha=alpha_flat_right[mask],

zorder=3,

)

for x_line in np.arange(-0.5, M, 1.0):

ax_r.axvline(x=x_line, c='black', linestyle='-', linewidth=1.0,

zorder=1)

for y_line in np.arange(-0.5, N, 1.0):

ax_r.axhline(y=y_line, c='black', linestyle='-', linewidth=1.0,

zorder=1)

ax_r.set_xticks(np.arange(M))

ax_r.set_xticklabels(data_right.index, rotation=90)

ax_r.set_yticks([])

ax_r.tick_params(axis='x', labelsize=7, length=2.5)

ax_r.margins(0, 0)

# ---------------------------------------------------------------------------

# legend

marker_kwargs = dict(

marker='o',

linestyle='none',

markeredgecolor='black',

markeredgewidth=0.4,

)

left_handles = [

Line2D([], [], markerfacecolor='#C4C4C4', markersize=3,

label='Absent', **marker_kwargs),

Line2D([], [], markerfacecolor='#d65db1', markersize=7,

label='Present', **marker_kwargs),

]

separator = Patch(visible=False, label=' ')

right_colours = ['#d65db1', '#ff6f91', '#008f7a', '#ffc75f']

right_labels = ['1', '2', '3', '4']

right_sizes = [20.0, 40.0, 60.0, 80.0]

right_handles = [

Line2D([], [], markerfacecolor=colour,

markersize=2 + 6 * size / 80,

label=label, **marker_kwargs)

for colour, size, label in zip(right_colours, right_sizes, right_labels)

]

leg_ax.legend(

handles=[*left_handles, separator, *right_handles],

loc='center', bbox_to_anchor=(0.5, -0.5),

ncol=len(left_handles) + 1 + len(right_handles),

fontsize=5.5, title_fontsize=6,

frameon=False, handletextpad=0.3, columnspacing=0.3,

)

Plot-misc enhanced implementation (26 function calls)

import pandas as pd

import numpy as np

import matplotlib.pyplot as plt

import matplotlib.lines as mlines

from matplotlib.patches import Patch

from matplotlib.gridspec import GridSpec

from plot_misc import incidencematrix as imat_plt

# ~~~~~~~~~~~~~~~~~~~~~~~~~~~~~~~~~~~~~~~~~~~~~~~~~~~~~~~~~~~~~~~~~~~~~~~~~~~~~

DOT_COLOUR = [('#C4C4C4', 0), ('#d65db1', np.inf)]

fig = plt.figure(figsize=(7*1/2.54, 6*1/2.54))

gs = GridSpec(nrows=2, ncols=2, width_ratios=[1, 1], height_ratios=[1, 10],

wspace=0.1, figure=fig)

ax_l = fig.add_subplot(gs[(1,0)])

ax_r = fig.add_subplot(gs[(1,1)])

leg_ax = fig.add_subplot(gs[0, :])

# Left panel

_ = imat_plt.draw_incidencematrix(table>0).astype(int), ax=ax_l,

dot_colour=DOT_COLOUR, dot_size=[8, 40], lw = [1,1],

tick_lab_size=[10, 10], tick_len=[5,5], margins=[0.08, 0.06],

kwargs_scatter_dict={'edgecolor': 'black', 'linewidths':0.4, },)

ax_l.tick_params(axis='y', labelsize=7, length=2.5)

ax_l.tick_params(axis='x', labelsize=7, length=2.5)

# Define cut-offs and mappings

DOT_COLOUR = [

('#AAAAAA', 0.9), # grey for (−inf, 0.2]

('#d65db1', 1.9),

('#ff6f91', 2.9),

('#008f7a', 3.9),

('#ffc75f', 4.9),

]

# Size thresholds: 2 categories

DOT_SIZE = [

(0, .9), # grey for (−inf, 0.2]

(20, 1.9),

(40, 2.9),

(60, 3.9),

(80, 4.9),

]

TICK_LAB_SIZE = (10, 10)

# Right panel

_ = imat_plt.draw_incidencematrix(data=table, ax=ax_r,

lw = [1,1], line_colour=['black', 'black'],

dot_colour=DOT_COLOUR, dot_size=DOT_SIZE,

dot_transparency=[1], tick_lab_size=TICK_LAB_SIZE,

tick_len = [0,0], margins=[0,0],

grid_position='outline',

kwargs_scatter_dict={'edgecolor': 'black', 'linewidths': 0.4},

)

ax_r.set_yticks([])

ax_r.set_yticklabels([])

ax_r.tick_params(axis='x', labelsize=7, length=2.5)

# left panel legend: binary absent/present

_SCATTER_KW = dict(marker='o', linestyle='none',

markeredgecolor='black', markeredgewidth=0.4,)

left_handles = [

mlines.Line2D([], [], markerfacecolor='#C4C4C4',

markersize=3, label='Absent', **_SCATTER_KW),

mlines.Line2D([], [], markerfacecolor='#d65db1',

markersize=7, label='Present', **_SCATTER_KW),

]

# right panel legend: score 0-4, colours and sizes match DOT_COLOUR / DOT_SIZE

_RIGHT_COLOURS = [c for c, _ in DOT_COLOUR[1:]]

_RIGHT_LABELS = ['1', '2', '3', '4']

_DOT_SIZE_VALS = [s for s, _ in DOT_SIZE[1:]]

right_handles = [

mlines.Line2D(

[], [], markerfacecolor=col,

markersize=2 + 6 * s / 80 if s > 0 else 2,

label=lbl, **_SCATTER_KW,

)

for col, s, lbl in zip(_RIGHT_COLOURS, _DOT_SIZE_VALS, _RIGHT_LABELS)

]

leg_ax.axis('off')

separator = Patch(visible=False, label=' ') # blank spacer

leg_ax.legend(

handles=[*left_handles, separator, *right_handles],

loc='center', bbox_to_anchor=(0.5, -0.5),

ncol=len(left_handles) + 1 + len(right_handles),

fontsize=5.5, title_fontsize=6,

frameon=False, handletextpad=0.3, columnspacing=0.3,

)
